# Appropriate Strategies for Reducing the Negative Impact of Online Reports of Suicide and Public Opinion From Social Media in China

**DOI:** 10.3389/fpubh.2021.756360

**Published:** 2021-12-03

**Authors:** Meijie Chu, Hongye Li, Shengnan Lin, Xinlan Cai, Xian Li, Shih-Han Chen, Xiaoke Zhang, Qingli Man, Chun-Yang Lee, Yi-Chen Chiang

**Affiliations:** ^1^State Key Laboratory of Molecular Vaccinology and Molecular Diagnostics, School of Public Health, Xiamen University, Xiamen, China; ^2^Department of Technical Cooperation, Zhiwei Research Institute, Beijing, China; ^3^School of International Business, Xiamen University Tan Kah Kee College, Zhangzhou, China

**Keywords:** online media, suicide reporting, public opinion, negative impact, China

## Abstract

Suicide events may have a negative impact on all of society. The media plays a significant role in suicide prevention. Therefore, the aims of this study are (a) to understand the association between characteristics of suicide events and characteristics of who committed suicide, and event impact indexes (EIIs) of suicide reported on the internet; (b) to analyze violation of recommendations for reporting suicide by Weibo, and (c) to investigate the effect of online reports of suicide on public opinion. We carried out a content analysis of online reports of suicide. This study analyzed 113 suicide events, 300 news reports of suicide, and 2,654 Weibo comments about suicide collected from the WeiboReach between 2015 and 2020. We used a *t*-test and analysis of variance (ANOVA) to explore the potential factors associated with the EIIs of suicide events. The results found that (a) The suicide events reported on the internet during COVID-19 and those related to celebrities and students tend to have higher EIIs; (b) suicide reports on Weibo frequently violated WHO recommendations for suicide reporting in the media; and (c) public opinion of suicide reporting in the online media was mostly emotional and irrational, which is not beneficial for public mental health and suicide prevention. In conclusion, first, the situation of many people working from home or studying from home and spreading more time online during COVID-19 may lead to suicide events obtain more public attention. Online media could further improve public responsible reporting and daily media-content surveillance, especially taking particular care in those suicide events during COVID-19, and related to celebrities and students, which may have a higher event impact on the internet. Second, health managers should regular assessment of observance of the WHO recommendations for suicide reporting by online social media to prevent suicide. Third, health communication managers should use big data to identify, assess, and manage harmful information about suicide; and track anyone affected by suicide-related reports on social media to reduce the negative impact of public opinion to intervene suicide in the early stage of suicide.

## Introduction

Suicide is now a major public health problem. According to the World Health Organization (WHO), every year, more than 700,000 people die by suicide; that's one person every 40 s. Suicide occurs in all regions of the world, especially in developing countries (1). Some developing countries, including China, have experienced rapid economic development and social change. Such developments have produced higher social competition and the acceleration of the pace of life, which has caused people's work pressure, study pressure, and life pressure to increase. Nearly 40% of suicides each year occur in China or India (2). In China, every 2 min, one person dies by suicide and 8 people attempt suicide (3). Suicides have a strong impact on families, communities, and societies. Nearly 1.5 million people suffer long-term and severe psychological trauma every year due to suicide by family members or friends (3). Taking appropriate strategies to reduce the negative impact of suicide and public opinion toward suicide events is critical to achieving the optimistic situation for suicide prevention.

Due to rapid digitalization, there is a recent surge in online media reports of suicidal events. Increased social media/internet use was associated with suicide attempts (4). A previous study found that media reporting of suicide was related to an increase in actual suicide (5). The media plays a significant role in suicide prevention. Low-quality suicide reporting may harm the public and even lead to copycat suicide behavior. Repetitive reporting about the same suicide events and the reporting of suicide myths lead to an increase in suicide rates (6). The phenomenon of media suicide reporting increasing the risk of suicide is known as the “Werther Effect.” The coverage of online media reports of suicide is extensive and prominent, and they may report suicide in an exaggerated and overly-detailed manner. These features make online reports of suicide receive more attention from the public, especially vulnerable individuals. According to social learning theory, the public may learn the solution of personal problems by suicide and then adopt suicidal behavior (7). The appropriate strategy for reducing the impact of harmful information about suicide events on netizens and enhancing the online media's responsibility for suicide reporting is needed for suicide prevention.

### Explore the Potential Factors Associated With the Communicate Effect of Online Suicide Events

Based on social cognitive theory, the four major subfunctions governing observational learning include attentional, retention, production, and motivational processes (8). The attentional process is the first step of observational learning. A previous study found that subsequent online suicide-related search behaviors may increase among the general population in the weeks following celebrity suicide (9). Suicide events with high communication effects would be are easier to be seen by the public, and then generate copycats. Based on agenda-setting theory, those suicide events with high influence levels can enter into the hot search list. Therefore, it is important to investigate the association between the characteristic of suicide events and the communication effect of suicide events on the internet.

Previous studies have found that the characteristics of suicide events, including the gender, age, and social identity of the deceased, as well as suicide method, suicide-related headlines were associated with copycat suicides (10, 11). The celebrity status of the deceased is associated with an increased risk of a post-report increase of similar suicides (10–12). Thus, this study focused on the effect of the social identity of suicide on the communication effect of online reported suicide-related events.

In addition, the COVID-19 pandemic has affected all people across the globe. Mental stress has become a serious global public health issue, accentuated by the COVID-19 pandemic (13, 14). Mental health symptoms in China during the COVID-19 pandemic are prevalent (15). Some studies found that there were increased event rates for suicidal ideation, suicide attempts, suicidal behavior, and self-harm during the COVID-19 pandemic (16, 17). Besides, many people work from home or study from home during the COVID-19 pandemic. We may spend more time on the internet. Therefore, this study explored whether the communication effect of online suicide events has increased during the COVID-19 pandemic.

The event influence indexes (EIIs) are authoritative indexes to portray the communication effect of a single event on the internet, which is based on social media and network media data for the whole network. The first aim of our study was to explore the potential factors associated with the event impact indexes (EIIs) of suicide reported on the internet.

### Analyze Violations of the WHO Media Guidelines of Suicide-Related Reporting

Internet use is also widespread in China. As of June 2020, the number of internet users reached 940 million, accounting for ~67% of the country's total population (18). The number of internet users was positively associated with suicide rates in the general population (19). In addition, mobile access has become widespread, and 99.2% of internet users use mobile phones to surf (18). The average online time per person reaches 28.0 h per week (18). The internet, especially smartphones, has become the main channel through which most people obtain news and information in China. Social media can make the public more sensitive to external stimuli (20). One study revealed that suicide prevention programs need to target internet users in China (21). A previous study has explored the responsibility of online suicide stories in China from 2003 to 2015 on some newspapers and some internet-based media sources (22). It is also critical to focus on analyzing the quality of suicide-related reporting in social media platforms in China.

Sina Weibo, which emerged in 2010, has been a leading Chinese online social network (OSN), just like Facebook in other countries. There were more than 516 million active daily users by the end of 2019. Sina Weibo has distinctive characteristics of online media. These users use Weibo functions (e.g., like, @, tick, forward, comment function) to interact with each other in real-time. Sina Weibo data are emerging as a key online medium and data source for researchers to understand social problems in a non-invasive way. As the number of netizens and Sina Weibo users in China has been increasing, this study would assess the use of media reporting recommendations in suicide news items during 2015–2020 in Sina Weibo. Therefore, the second aim of this study was to analyze violations of the WHO media guidelines of suicide-related reporting by Sina Weibo.

### Investigate Public Opinion Toward Online Reports of Suicide

The situation of suicide topics discussed and communicated in the online media may lead to public negative moods increase, and more people obtain information about suicide methods (23). We need to explore measures to avoid this bad situation. The internet provided new channels for news reporting and created opportunities for the public to express their opinions and attitudes instantly through readers' comments (24). People express a higher degree of their attitude and emotion in social media than in face-to-face settings (25, 26). Therefore, analyzing readers' comments on online suicide-related news reports can provide valuable information about people's opinions and emotions.

At present, the public opinion of online media in China has the characteristics of original content, shock effects, zero cost, and diversification of subject and word expression (27, 28). The benefit of public opinion topics and the polarization effect make it easy for online public opinion to influence actual personal behaviors. If these behaviors are negative, they may cause very serious consequences to society. Sina Weibo can set the top trending searches according to the level of attention of the event. These events that arouse more attention and discussion will be ranked in the top rank of searches to attract wider attention and discussion, thereby generating a strong public opinion effect. However, few studies focus on the public opinion of online reports of suicide in Weibo in China.

Suicides among different social identities may have a varied influence on the subsequent suicides and public emotions. Unfortunately, it is unclear how the public's expression of social, emotional, and cognitive psychological responses to reports of suicide differ based on the social identities of the deceased. Thus, the third objective was to investigate the negative effect of public opinion toward online reports of suicide across different social identities of the deceased.

## Materials and Methods

### Data Source and Procedure

#### The Process of the Collection of Online Reported Suicide-Related Events

Data were derived from the Harbin Institute of Technology Joint Laboratory for social networks and a data mining research team named WeiboReach, which is one of the most advanced new media (including Weibo, Wechat, and other online media) news propagation analysis tools in China.

To obtain online reported suicide-related events, this study firstly determined keywords. The people who committed suicide, the location of the suicides, and the cause of suicides are always various, while the methods of suicide are common. When reporting suicide-related events, people may pay attention to the method and then lead to copycat suicide. Thus, the keywords in this study included the word suicide itself and other keywords derived from suicide, and many suicide methods. The suicide methods referred to a previous study and an integrated national mortality surveillance system for death registration and mortality surveillance in China (29, 30). Keywords in this study included “suicide, commit suicide, exclusion of homicide, hanging, charcoal burning, jumping, high fall, slitting wrists, sleeping pills, drugs, cutting the neck, jumping into the sea and throwing into the lake.” These keywords were related to suicidal behavior that was commonly reported in the news. This study applied these keywords in Chinese to obtain events that contain these keywords in the event titles in the WeiboReach database from 2015 to 2020 by a keyword maximization search method. WeiboReach database contains popular, classic, and influential events in the Chinese public opinion environment. Those events included corporate events, social events, and other types of events data. Finally, a total of 175 suicide-related online events were retrieved from 2015 to 2020 in the WeiboReach database.

#### Selecting Online Suicide Events for Analyzing Potential Factors Associated With the EIIs

This study included a total of 113 suicide events after excluding duplicate events and non-suicide events. We explored the potential factors (including characteristics of suicide events and characteristics of who committed suicide) associated with the EIIs among the 113 suicide events by single-factor analysis.

#### Collecting Suicide News to Understand the Situation in Violation of Recommendations for Reporting Suicide

We ranked the 113 online suicide events by EII. The top 30 suicide events with the EII more than 65 can include as many social identities of people who committed suicide as possible, such as celebrities, officials, entrepreneurs, students, and the general population. Since the EII of an event exceeds 65, some institutions would perform emergency management for this event (31). Therefore, the top 30 suicide events were selected for further detailed analysis.

According to the “top 10 effects” of public opinion in the cyberspace of the mainland of China, the opinions and comments of the top 10 netizens determine dozens or even hundreds of subsequent opinions and comments on certain news or social phenomena on the internet, thus forming an online public opinion (32). This study collected the top 10 most popular Sina Weibo news items for each of the 30 suicide events with high EII, based on a combination of web crawler for the Sina Weibo platform and Sina Weibo API (Application Programming Interface). Therefore, a total of 300 Sina Weibo suicide-related news reports were included. We analyzed the current situation in non-observance of the six do nots and observance of the six dos in Weibo suicide-related news reporting.

#### Collecting Weibo Comments to Analyze Public Opinion

**Figure 1:**
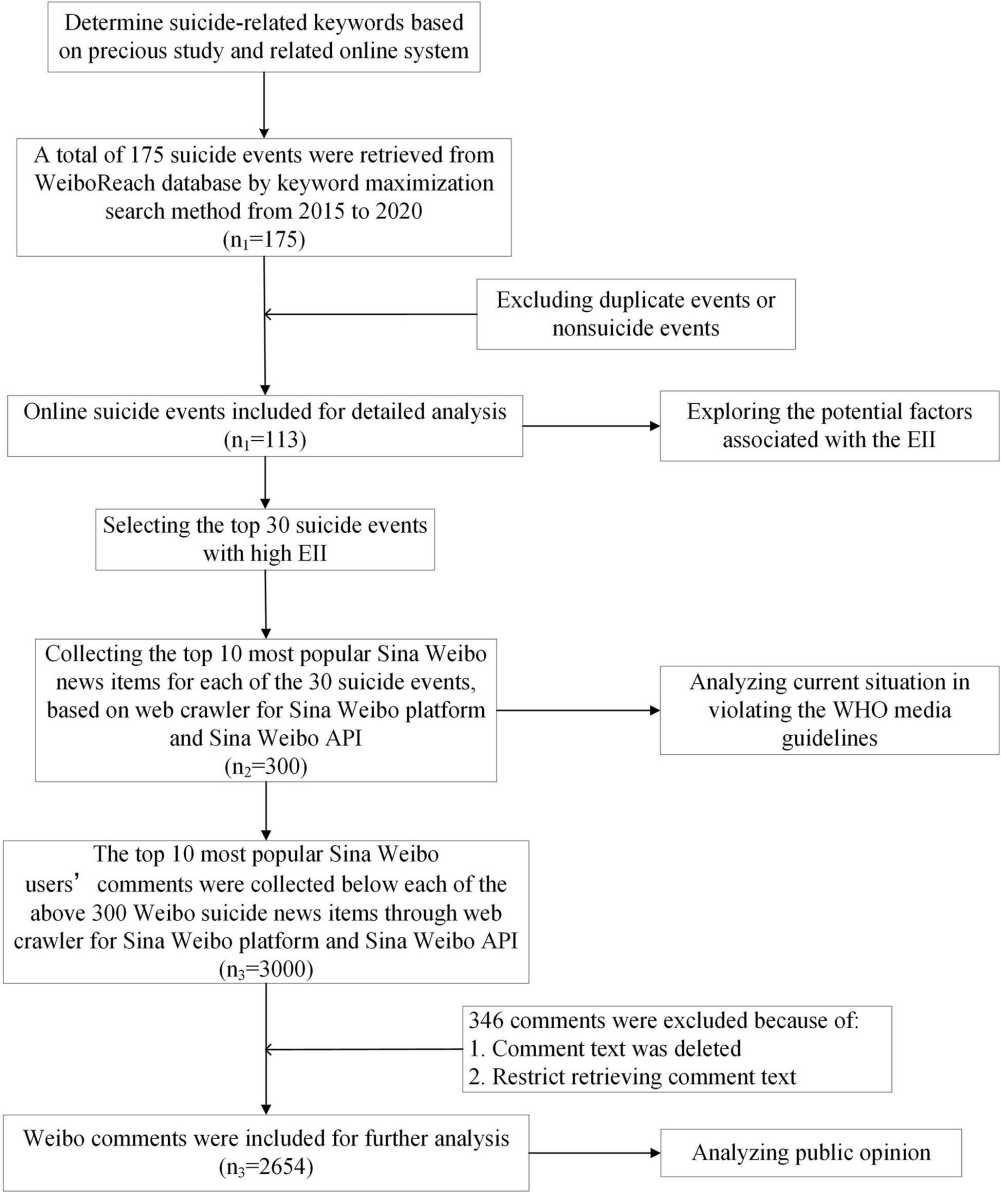
Flow chart of the study. EII, Event Influence Index.

The top 10 most popular Sina Weibo users' comments were collected below each of the above 300 Sina Weibo suicide-related news items, based on the combination of web crawler for Sina Weibo platform and Sina Weibo API. Since some comments were deleted and could not be retrieved, a total of 2,654 comments were included in this study. We further analyzed public opinion by different social identities of the deceased using quantitative content analysis. Figure 1 shows the flow diagram used in our study.

### The Measurement of the Event Influence Indexes

The EIIs can be used to portray the communication effect level of events. The communication effect level was calculated by taking into account the position of the keywords (i.e., Weibo headline or Weibo posts content), the weight of communication channels (i.e., Weibo, WeChat, and other online media), and the weight of event attributes. The communication effects of events on social media and online media were summarized, and then, the event influence was normalized to obtain the EIIs, which ranged from 0 to 100. One study has verified the effectiveness of the method of quantifying online events dissemination (33). The previous study also used EII to understand the communication effect level of events (31).

### Data Analysis

First, content analysis was conducted on 113 online suicide events reporting text since it analyzes qualitative data by statistical formalism. The researchers carried out coding of suicide reports based on a preliminary draft coding manual. The coding involved the characteristics of suicide reporting, including time of suicide, first news release channel, the identity of first reporting on Weibo, the social identity of people who committed suicide, whether committed suicide was a student, gender of the deceased, age of the deceased, whether others were injured, suicide method, suicide location. After conducting descriptive statistics on the abovementioned data, we also carried out an analysis of variance (ANOVA) and *t*-tests to explore the relationship between different characteristics of suicide and the EIIs of suicide. Second, we further coded violations of the WHO media guidelines for 300 suicide-related news items between 2015 and 2020. Then, we calculated the violation rate, which was defined as the proportion of the number of news items violating the guidelines. Finally, to analyze the effects of suicide-related reporting on public opinions, we organized the psychological impact and coping patterns of netizens for the social identities of those who committed suicide using Weibo comments. The social identities of celebrities accounted for 10 of the 30 suicide events analyzed, students accounted for 9, and general people accounted for 11.

We used Microsoft Excel (Microsoft Corporation) and SPSS software 21.0 (SPSS Inc.) for statistical analyses, and *P* < 0.05 was considered significant.

## Results

### The Association Between Characteristics of Suicide Events and Characteristics of Who Committed Suicide, and Event Impact Indexes

Table 1 shows the relationship between characteristics of suicide and EIIs of suicide events reporting on the internet. The characteristics of suicide events in our study included event time, first releasing channel, cause other deaths or victims, suicide method, suicide site, and identity of the first reporter on Weibo. Of the 113 suicide events, 74 occurred before the COVID-19 pandemic, with a mean value of 58.52 for the EII, while 39 occurred during the COVID-19 epidemic, with a mean value of 63.88 for the EII. The EII was significantly higher during the COVID-19 epidemic than before (*P* = 0.017). The relationships between other characteristics of suicide events and EIIs of suicide events were not statistically significant.

**Table 1:** Relationship between characteristics and EII of suicide reports on the internet (n_1_ = 113 suicide events).

	**No. of events**	**Mean of EII**	***t* or *F***	***P***
**Characteristics of suicide events**				
**Events time**^**c**^			2.415^a^	0.017^*^
Before COVID-19	74	58.52		
During COVID-19	39	63.88		
**First releasing channel**			−1.285^a^	0.202
Weibo	29	63.14		
WeChat or other online media	84	59.41		
**Other deaths or victims caused by suicide deceased**			0.385^a^	0.701
No	89	60.11		
Yes	24	61.31		
**Suicide method**			0.560^b^	0.573
Falling	48	59.90		
Hanging	15	63.82		
Others	50	59.77		
**Suicide site**			1.117^b^	0.331
Home, hotel, apartment, rental house	31	62.72		
School	15	62.53		
Others	67	58.79		
**Identity of the first reporter on Weibo**			0.432^a^	0.668
Blue V	73	63.71		
Yellow V	32	62.65		
**Characteristics of who committed suicide**				
**Social identity**			4.598^a^	0.012^*^
Celebrity	37	64.47	(C, S > G)
Student	25	62.57		
General people	51	56.31		
**Gender**			0.003^b^	0.997
male	58	60.40		
female	41	60.25		
Unknown or multi-person	14	60.57		
**Age**			0.377^b^	0.687
Up to 44 years old	76	60.24		
45~59 years old	17	58.54		
Unknown or multi-person	20	62.39		
**Country**			−1.257^b^	0.211
China	94	59.65		
Others	19	63.92		

a *The value of t*,

b *The value of F*;

c *As for the events time, if online reported suicide events occurred from January 1, 2015, to December 30, 2019, was defined as “Before COVID-19”; and events occurred after December 31, 2019, was defined as “During COVID-19.”*

* *P < 0.05. We used post-hoc tests by Student-Newman-Keuls (SNK). Blue V represents the official authority, which is a logo that can only be certified by large organizations such as governments, businesses, schools, and media. Yellow V represents individual bloggers with more than 10 million readers per month*.

In addition, the characteristics of those who committed suicide included their social identity, gender, age, and country. The mean value of the EII for suicide events reporting the social status of the deceased as a celebrity was 64.47, while the mean value of the EII for students was 62.57 and the general population was 56.31. The social identity of the deceased was related to the EIIs of suicide events (*P* = 0.012). The results of *post-hoc* tests showed that the EII of suicide events related to celebrities and students was higher than that of the general population. Besides, there were no statistically significant associations between other characteristics of who committed suicide and EIIs of suicide events.

In summary, the suicide event time, and the social identity of the deceased were significantly associated with EII. The suicide events reported on the internet during COVID-19 and those related to celebrities and students tend to have higher EII.

### The Rate of Online Reports of Suicide Violating the WHO Media Guidelines Between 2015 and 2020

**Figure 2:**
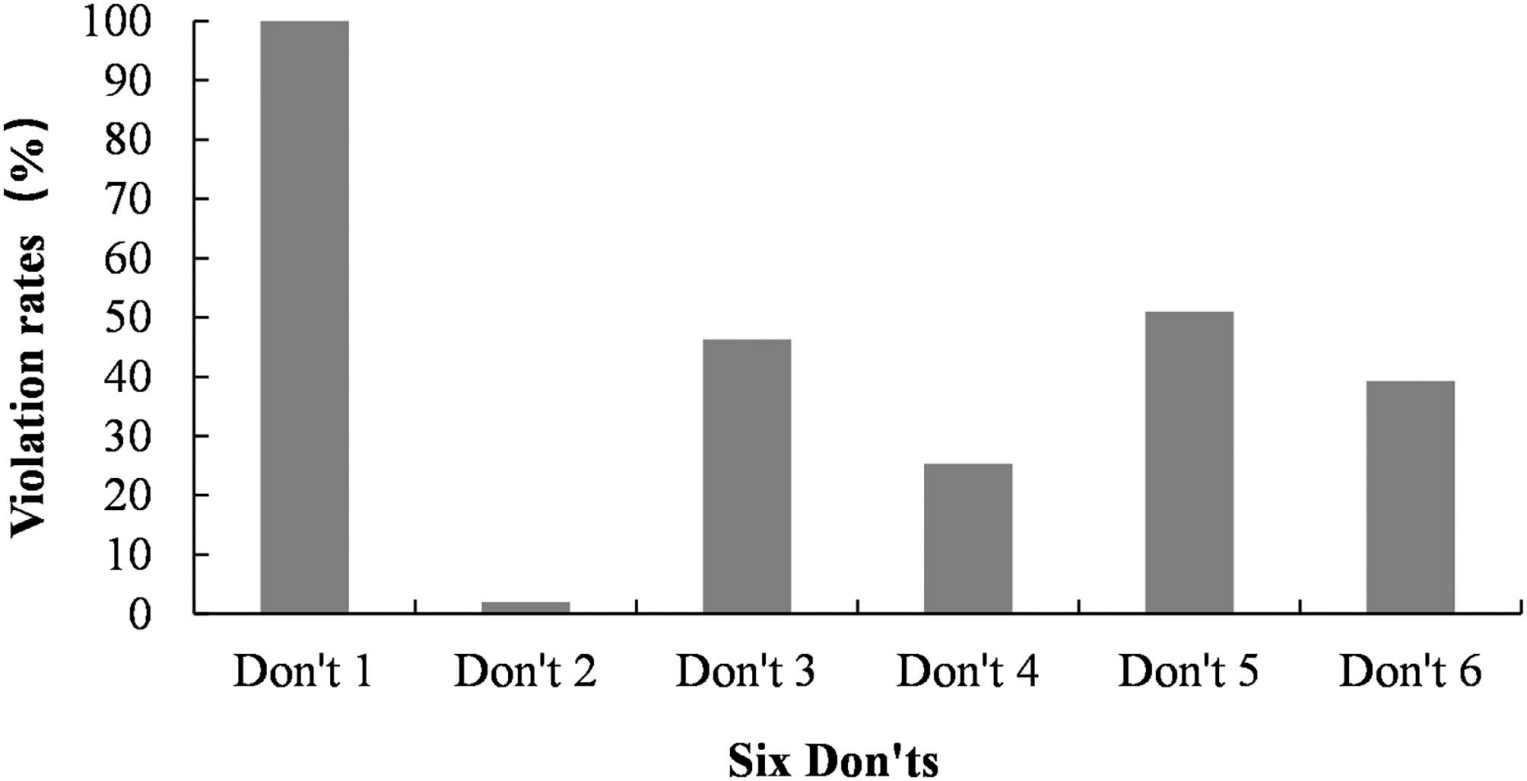
Violation of the WHO recommended list of suicide reporting on Weibo (n_2_ = 300 Weibo news items). Don't 1: Avoid prominent placement and unique repetition of stories about suicide; Don't 2: Avoid language which sensationalizes or normalizes suicide, or presents it as a solution to problems; Don't 3: Avoid explicit description of the method used in a completed or attempted suicide; Don't 4: Avoid providing detailed information about the site of a completed or attempted suicide; Don't 5: Avoid sensational headlines; Don't 6: Avoid using photographs, unedited video footage, or links to electronic media.

Figure 2 shows the proportion of violations of the WHO media guidelines by Weibo news. Violations of don't number 1 (Avoid prominent placement and unique repetition of stories about suicide) were most severe. The number 1 don't was observed in all 300 Weibo suicide-related news items. Approximately 51% of the online reports violated the number 5 don't of “Avoid sensational headlines.” Some of these reports included the word suicide in the headline, and others included the suicide method and the site of a suicide. Approximately 46.3% of Weibo news violations were related to don't number 3 (Avoid explicit description of the method used in a completed or attempted suicide), and 39.3% of violations were related to don't number 6 (Avoid using photographs, unedited video footage or links to electronic media).

In terms of the rates of violating the WHO list of dos for suicide reporting by Weibo news, the results showed that none of the news items observed the number 1 do (Provide information about where to seek help), number 2 do (Take the opportunity to educate the public about suicide and suicide prevention, without spreading suicide-related gossip) or number 3 do (Provide information on how to cope with life stress or suicidal thoughts and on helplines). Forty percent of the celebrity stories violated number 4 do (take particular care in reporting celebrity suicides). These Weibo reports described the site and method used in suicides among celebrities.

### The Public Opinion Toward Online Reports of Suicide Events

**Figure 3:**
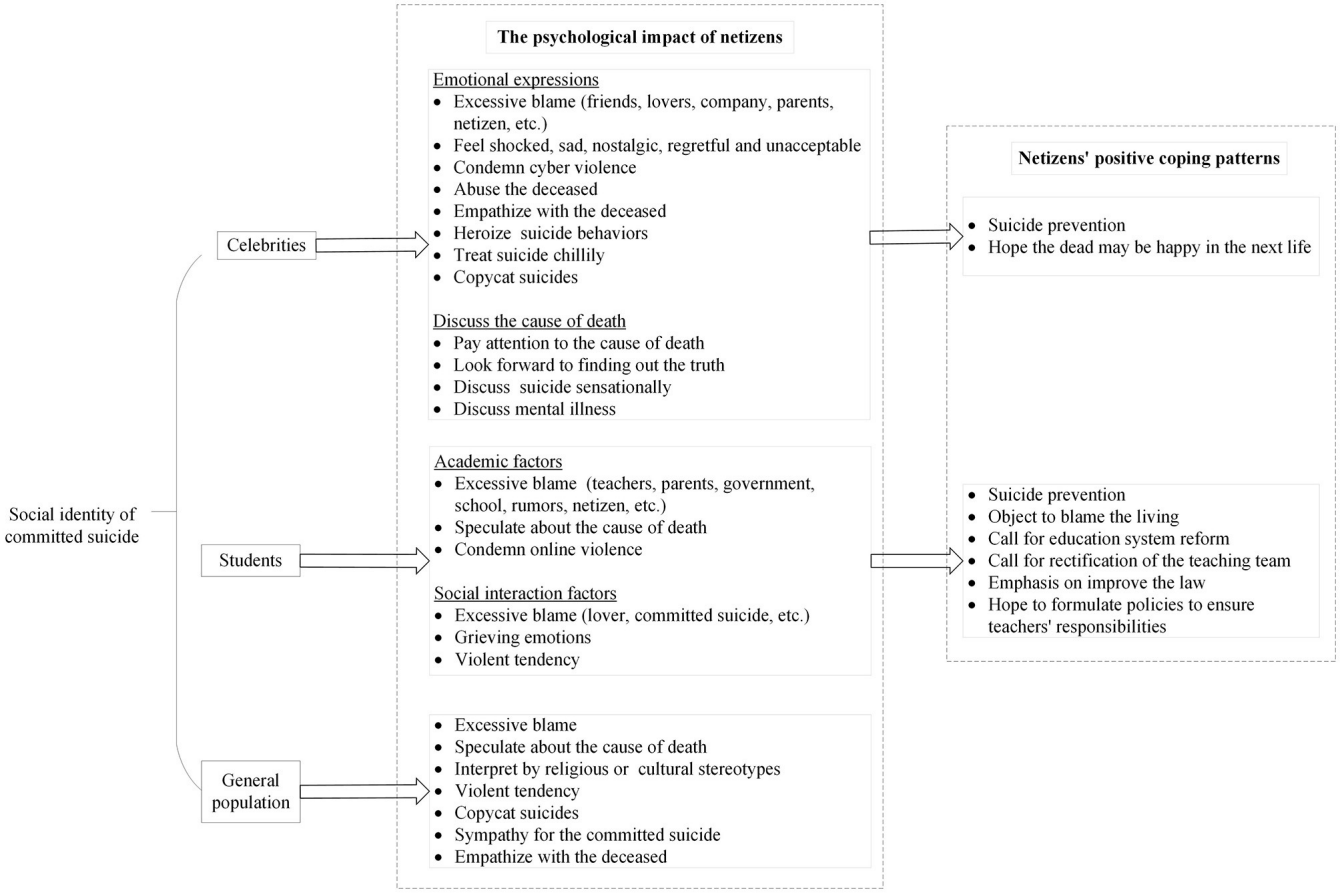
The effect of high EII suicide-related news on public opinion and response patterns of netizens by different social identities (n_3_ = 2,654 Weibo comments).

To understand the public opinion toward suicide events based on different social identities of the deceased, we conducted text content analysis using Weibo comments below the top 300 most popular Weibo news items of 30 suicide events with higher EII. Figure 3 shows the effect of high EII suicide-related news on public opinion and positive coping patterns of netizens by different social identities of the deceased, such as celebrities, students, and the general population. This study found that suicide reporting raised great public attention and negative sentiment on the internet in China. Netizens showed excessive blame for the deceased, people bereaved by suicide, and others related to committed suicide and discussed the cause of death regardless of the social identity of the deceased.

When the deceased person was a domestic or international celebrity, netizens feel shocked, sad, nostalgic, regretful, and unacceptable. Some people empathize with the deceased. People may sensationalize or normalize suicide online reports of suicide among celebrities. Suicide among celebrities makes some people heroize suicide, which in turn can lead to copycat suicides. If the deceased is an official and the cause of suicide is related to work, netizens would be interested in the cause of death and expect to determine the truth.

The public tends to object to blame the teachers, parents, and schools of the deceased, and they may even blame the government, rumors, and netizens. In response to comments blaming teachers, netizens condemned online violence. If Weibo published news about a student dying by suicide due to social interaction, the public blames the deceased and their lovers. Public opinion tended toward grief and violence.

In terms of suicide events among the general population, netizens sympathize with people bereaved by suicide and innocent people who are victims or dead caused by the suicide event. For childhood suicide due to family factors, people sympathize with the deceased and blame their guardians. Netizens also posted extreme and violent comments. Moreover, people may interpret suicidal behavior through the lens of religious or cultural stereotypes. Online reports of suicide may lead to suicidal ideation among the general public.

Overall, the majority of public opinions about online reports of suicide were emotional and irrational, which are not conducive to the psychological health of netizens. However, when committed suicide is a celebrity, netizens develop positive coping patterns to prevent suicide. Netizens actively call for suicide prevention and hope that they will be happy in the next life. If the media reports that a student's suicide was due to academic factors, netizens would post comments that favor suicide prevention and suggest that suicide is not a solution to problems. Some netizens also call for education system reform, improvement of the law, and hope to formulate policies to ensure the virtue and responsibilities of the teachers.

## Discussion

To understand how to reduce the negative impact of suicide-related reporting and public opinion, this study not only investigated the associated factors with the impact index of suicide events but also assessed the quality of suicide-related reporting by indicators of harmful information and helpful information according to the WHO guidelines by Weibo using suicide events with high EIIs. Then, we further explored the specific connotation of online public opinion about suicide-related reporting and the unique psychological impact of current emotional and irrational online public opinion on the public by analyzing big data on Sina Weibo users' responses.

### Focusing on Reducing the Communication Effect of Suicide Reports and Paying More Attention to Vulnerable People During COVID-19

Traumatic events such as the current COVID-19 pandemic undoubtedly affect all demographics of people. People were more sensitive to negative events during critics. A previous study found that several anticontagion policies were associated with search interest levels for the term “suicide” in the online media (34). This study found that the impact indexes of suicide events were higher during COVID-19 than before COVID-19 the pandemic. The event impact indexes could portray the communication effect of an event on the internet. Thus, this study verified that online suicide events spread more widely during COVID-19. The current COVID-19 pandemic has the potential to cause mental instability. During the pandemic, people have been experiencing psychiatric disorders, such as fear (35, 36), stress, anxiety (37), disappointment, irritability, depressive disorders, and even suicide (38). The COVID-19 pandemic also has a dynamic impact on suicide probability (39). This study indicated that we could take steps to reduce the spread of suicide reports during COVID-19. Health communication professionals also need to pay more attention to vulnerable people with psychiatric problems during the COVID-19 pandemic.

### Taking Particular Care in Reporting Celebrity and Student Suicides

As for the relationship between characteristics of who committed suicide with the communicate effect of online reported suicide events, this study found that celebrity suicides were often regarded as being high impact indexes. The result was consistent with a previous study (40). Celebrity suicides are newsworthy, and reports of these events are more widespread. The celebrity status of the deceased tends to be an important driver of online engagement (41). Based on vertical identification theory (42), people tend to identify with those celebrities. In particular, suicide by entertainers with a large fan base has been found to draw more public attention and then trigger copycat effects. In addition, this study also found that student suicide generated more influence levels on the internet. The media prefer to report suicides among younger individuals and underreport suicides among those in late life (43). Suicide is the fourth leading cause of death among 15- to 29-year-olds globally (1), making it a serious global public health problem. Student suicides lead to a huge shock among families, schools, and society. However, only suicides generating a large amount of media coverage and a strong reaction on social media were followed by an increase in suicide rates (26, 44). To reduce the negative impact of online reports of suicide on the community, media professionals should take particular care in reporting celebrity and student suicides.

### Improving Responsible Suicide Reports and Providing Suicide Prevention Measures on the Social Media Platform

The WHO has provided a resource for media professionals about how to report on suicide (45). Previous studies found that online reports of suicide had low adherence to the responsible suicide reporting WHO guidelines (46–48). The results also found that the content of suicide-related news was not sufficiently appropriate, with all of the news failing to adhere to the WHO's six dos and six don'ts of suicide-related reporting. Some of these stories provide detailed information about the site of a suicide and explicit description of the method used in a suicide or suicide attempt by using photos and video footage. Some news even uses photos of the deceased without mosaics, and others report on suicide notes and social media posts of the deceased.

While the violation rates of don't No. 1 (Avoid prominent placement and unique repetition of stories about suicide) in this study were higher than previous results. Perhaps this is because we focused on suicide events with high impact, and we selected the top 10 news reports of each suicide event on Weibo. However, these reports are the ones that people focus on. There may be less proportion of suicide news stories violating the don't No. 5 (Avoid sensational headlines) of the WHO recommendations during 2015–2020 than during 2003–2015 (22). More coverage of online suicide-related news and suicide methods can lead to more opportunities to learn about suicide experiences by the public, and inappropriate content of suicide-related news can have a negative impact on suicide prevention. Regarding online suicide reports, we emphasized that media should not use photographs or video footage of the scene. While these media should word headlines carefully, and optimize algorithms to reduce the frequency of suicide events being settled as most searched hashtags.

According to agenda-setting theory, media can shape the public's perception of the importance of different issues by highlighting certain issues while downplaying others (49). The content and direction of the top information on the search engine may influence their attitude and behavior. This and a previous study found that helpful reporting practices were very limited (48). Therefore, media professionals could further prioritize suicide prevention issues to highlight problem-solving. That is, media strengthen the real-time gatekeeping of suicide-related news to downplay the negative impact of suicide reports and provide suicide help resources to encourage those at risk of suicide to seek help, such as mental health lines and contact information of social help agencies. Effective regulation and monitoring of suicide reporting appeared to have a positive impact on suicide prevention (50). We recommend that the social media platform establish an early suicide risk warning system with the functions of information warning, regular monitoring, and reporting, providing suicide intervention measures, preventing recurrence, etc. When recognizing suicidal thoughts, the platform can pop up a window or send a private message to provide suicide prevention measures, thereby improving the suicide prevention literacy of netizens.

### Appropriate Strategies for Reducing the Negative Impact of Public Opinion From Social Media

Previous studies showed that suicide survivors experience severe mental health consequences and grief reactions, such as higher levels of blaming and a need to conceal the cause of death, rejection, and shame (51). This study focused on suicide events comments posted by the general population. The results showed that the majority of the public opinions were negative, and helpful guidance for suicide prevention was limited. This study also found the comments about suicide-related news of netizens were emotional, irrational, and violent. Besides, reproachful responses of the netizens were common in our study. Therefore, in terms of suicide events with more communicate effect on the internet, online social media should use true, objective, and comprehensive reports to trigger people thinking to reduce the speculation of the cause of suicide and then form rational and harmonious public opinion.

Both the national internet supervision platform and companies should take responsibility to conduct regular monitoring of suicide reports and public opinion, such as deleting harmful statements in a timely manner and minimizing the spread of negative information. At present, for one criminal case, the Weibo platform closed the wrongly oriented super talk, disbanded the offending groups, resolutely curbed irrational behavior, and resolutely dealt with extreme speech to maintain the ecological balance of the online community. In addition, the Weibo platform banned and permanently closed the accounts of those who violated the cyber-provision, such as provoking trouble, attacking government agencies, malicious marketing, etc. We think that suicide reports and public opinions regarding suicide events that are not conducive to suicide prevention should also be taken and dealt with seriously.

### Advocating Positive Copying Patterns and Reducing Negative Public Opinion, Especially for Suicides of Celebrities and Students

Different social identities of deceased people generated various public opinions. One study showed that different careers of the deceased resulted in significant differences in emotional response, with entertainment careers creating more emotional responses (52). Public attitudes toward celebrity suicide and general people suicide differed (53). The results of our study also demonstrated that comments to a celebrity suicide revealed more emotional words, such as shocked, sad, nostalgic, regretful, and unacceptable. This was consistent with other studies (54, 55). Our study proves the accuracy and adaptability of social learning theory and vertical identification theory using public comments on celebrity suicide reporting on Weibo. Given the high social status of celebrities, some people may glorify celebrity suicide and view suicide as a sign of heroic behavior, which makes the task of suicide prevention more challenging.

However, these suicide reports of cerebritis could raise positive psychological coping patterns, which was helpful for suicide prevention. Evidence of this phenomenon was also revealed in another study; celebrity suicide-related news reports not only evoked negative, internalized emotions but were also related to the expression of positive emotions of the users (54). In our study, the results also indicated that when committed suicide is a student, netizens also expressed opinions on public affairs with some system reforms and suicide prevention. We thought that the government should take some system reforms according to the netizens' appeal into consideration to reduce the suicide rates. In addition, we recommend that health communication managers focus on the improvement of public risk awareness and popularizing positive psychological reactions during information dissemination to promote the success of suicide prevention programs.

### Theoretical Contributions to Suicide Prevention From a Public Health Perspective

This study could also provide some implications from a public health perspective. The results in this study indicated that the suicide events reported on the internet during COVID-19 and those related to celebrities and students tend to have higher EII. A single event with high EIIs represents that it has a widespread effect communication effect on the internet. Based on social cognitive theory, people get motivated through several processes including attention, retention, and production (8). For suicide death to occur, a person must have sufficient capability to enact a suicide attempt. A previous study also found that the frequency of exposure to negative events was associated with increased capability (56). When the suicide events spread more widely on the internet, it may have more impact on netizens' suicide ideas and suicide attempts. The one-to-many transmission dynamics characterized by the mass media were shown to generate copycat suicides (57). However, people may increase social media usage during the COVID-19 pandemic (58). This study proved that the communication effect of suicide events was high during the period of the pandemic prevention and control period. Thus, it is important to take measures to reduce the negative influence of suicide events for suicide prevention during COVID-19 and other crises or pandemics. Thus, it is important to take measures to reduce the negative influence of suicide events for suicide prevention. Health managers could supervise social media platform to carefully report on suicide events, especially for those suicide events during COVID-19, and those suicide events related to celebrities and students.

In addition, a previous study found Sina suicide Weibo users tended to express negative emotion-related words, anger-related words, sadness, or death-related word than Sina Weibo users without suicide ideas (59). This study also found the public opinion of suicide events was emotional, irrational, and violent. Thus, health communicators should take appropriate strategies for reducing negative public opinion, and track anyone affected by online reports of suicide on social media constantly to intervene in suicide in the early stage of suicide. Besides, some studies focused on analyzing the word expressing of suicide survivors and those people with suicide ideas (59, 60), while our study discussed the negative impact of suicide-related online reports from the perspective of public opinion. This study would provide theoretical contributions to suicide prevention. In order to intervene suicide in the early stage of suicide, it is recommended to set up a special agency to conduct unified management of public opinion in conjunction with relevant departments such as public health, health communication, and public security. When extreme negative emotions and suicide comments are monitored, the public security and platform managers need to locate the speaker's IP under the premise of protecting their privacy rights, to block impulsive suicide and suicide followers as soon as possible.

## Strengthens and Limitations

This study conducted text content analysis to understand the association between characteristics of suicide and the communication effect of suicide events, assess the quality of suicide-related reporting on online social media and explore public opinion on suicide-related reporting. We proposed appropriate strategies for health communication practitioners to take regulatory actions to prevent suicide and improve public perception and literacy of suicide prevention, thereby reducing suicide rates and preventing suicide in the early stage.

Although this study has made several contributions to the existing research, there are several limitations to this work. First, not all respondents in China were registered as members of the social media platform, which limits the possibility of generalization. Second, this study focused on the Sina Weibo platform to analyze the quality of suicide-related reporting and public opinion, which may lead to selection bias of suicide-related news and public reactions. However, Sina Weibo has been a leading Chinese online social network. This study can discuss the theoretical contributions of Sina Weibo to reducing the negative impact of suicide reporting. The violation of suicide reporting guidelines on other social media platforms also needs to be monitored. The researchers could use other social medias to understand public opinion toward reports of suicide events in the further. Third, this study only analyzed the content of suicide-related reports and did not investigate readers. Further study may conduct interviews or cross-sectional surveys of netizens to understand the impact they receive from reports of suicide and their attitude to suicide events of different social identities of the deceased people. Finally, we searched online reports of suicide and Sina Weibo comments from 2015 to 2020 to explore the quality of online suicide reports and public opinion in recent years. Future studies could use data about online suicide reports before 2015 from Sina Weibo and other social media platforms to understand how to reduce the negative impact of suicide events and public opinion, thereby reducing suicide rates.

## Conclusion

This study conducted quantitative research to understand the influence of the communication effects of suicide events and the quality of suicide reporting and used text content analysis to explore public reactions to social media in China. We found that the observance of six don'ts was common, while helpful information was limited. The event impact index of celebrity and student suicide events was greater than that of suicide events among the general population, and the suicide-related reporting during the COVID-19 pandemic was greater than before the COVID-19 pandemic. Public opinion of suicide reporting in the online media mostly was emotional and irrational. Social media lacked public opinion guidance in favor of suicide prevention. We recommend promoting responsible suicide-related reporting in the social media environment worldwide. In addition, health communication practitioners should provide ways to address emerging public concerns after suicide events to avoid the spread of negative emotions and copycat suicides. Practically, the findings of this study can serve as the baseline for the design of intervention strategies, for instance, more discussion about the scene of improving suicide-related reporting on social media to reduce the negative impact of online reports of suicide and public opinion.

## Data Availability Statement

The datasets used and/or analysed during the current study are available from the corresponding author on reasonable request.

## Author Contributions

Y-CC has full access to all of the data in the study and take responsibility for the integrity of the data and the accuracy of the data analysis. Y-CC, C-YL, MC, and HL conceived of the study, participated in its design, and coordination and drafted the manuscript. QM performed the data acquisition and sampling. HL, SL, XC, XL, S-HC, and XZ performed the data analysis. All authors approved the final version and all take responsibility for its content.

## Funding

This paper was funded by the Fujian Provincial Social Science Foundation project “Empirical Research on Mental Health Promotion in the New Era of Healthy Aging” (Project Number: FJ2021T009) and the Scientific Research Grant of Fujian Province of China (No. Z0230104).

## Conflict of Interest

QM was employed by Zhiwei Research Institute. The remaining authors declare that the research was conducted in the absence of any commercial or financial relationships that could be construed as a potential conflict of interest.

## Publisher's Note

All claims expressed in this article are solely those of the authors and do not necessarily represent those of their affiliated organizations, or those of the publisher, the editors and the reviewers. Any product that may be evaluated in this article, or claim that may be made by its manufacturer, is not guaranteed or endorsed by the publisher.
